# METTL14 promotes migration and invasion of choroidal melanoma by targeting RUNX2 mRNA via m6A modification

**DOI:** 10.1111/jcmm.17577

**Published:** 2022-10-20

**Authors:** Xi Zhang, Xiaonan Zhang, Tengyue Liu, Zhe Zhang, Chiyuan Piao, Hong Ning

**Affiliations:** ^1^ Department of Ophthalmology The First Hospital of China Medical University Shenyang Liaoning China; ^2^ Department of Urology The First Hospital of China Medical University Shenyang Liaoning China

**Keywords:** choroidal melanoma, methyltransferase‐like 14, N6‐methyladenosine, runt‐related transcription factor 2, Wnt/β‐catenin signalling

## Abstract

The modification of N6‐methyladenosine is involved in the progression of various cancers. This study aimed to clarify its regulatory mechanism in the pathogenesis of choroidal melanoma. Expression of methyltransferase‐like 14 in choroidal melanoma or normal choroidal tissues was determined by Western blot and immunohistochemistry. The impacts of methyltransferase‐like 14 on invasion and migration of choroidal melanoma cells were determined using functional and animal experiments. The interaction between methyltransferase‐like 14 and its downstream target was identified by methylated RNA immunoprecipitation and a dual‐luciferase reporter assay. Additionally, Wnt/β‐catenin signalling pathway was evaluated by Western blot. Methyltransferase‐like 14 was upregulated in choroidal melanoma compared to the normal choroidal tissues. Overexpression or knockdown of methyltransferase‐like 14 enhanced or inhibited the invasion and migration of choroidal melanoma cells, respectively, both in vivo and in vitro. Methyltransferase‐like 14 directly targeted downstream runt‐related transcription factor 2 mRNA, depending on N6‐methyladenosine. Additionally, the Wnt/β‐catenin signalling pathway was activated by methyltransferase‐like 14 in choroidal melanoma cells. Our study identified a novel RNA regulatory mechanism in which runt‐related transcription factor 2 was upregulated by enhanced expression of methyltransferase‐like 14 via N6‐methyladenosine modification, thus facilitating migration and invasion of choroidal melanoma cells.

## 1 INTRODUCTION

Choroidal melanoma (CM) is an intraocular malignancy commonly occurring in adults, with 90%–95% of cases arising from the choroid.^1^ CM can potentially metastasize to various organs/tissues, including the liver, soft tissues, bone, lung, skin and lymph nodes.^2^ The prognosis of CM is characterized by high rates of mortality and metastasis; however, the mechanism of metastasis in CM is unclear.^3^

N6‐methyladenosine (m6A) is a widely observed modification in various cancers, and abnormal m6A modifications are associated with tumour progression.^4^, ^5^, ^6^ Liu et al. reported that mutations in methyltransferase‐like 14 (METTL14) or downregulation of METTL3 reduced the m6A modification of genes related to the AKT pathway in endometrial cancer. As a result, the AKT signalling pathway was activated, promoting tumorigenesis.^7^ Yu et al reported that insulin‐like growth factor 2 mRNA‐binding protein 2 (IGF2BP2), an m6A reader in RNA, stabilizes Slug mRNA and that this effect is dependent on m6A. IGF2BP2 showed a positive correlation with epithelial–mesenchymal transition as well as lymphatic metastasis of head and neck squamous cell carcinoma (HNSCCs).^8^ Li et al. demonstrated that DIAPH1‐AS1 m6A methylation mediated by WTAP was involved in nasopharyngeal carcinoma tumorigenesis and metastasis.^9^ Thus, m6A is closely associated with cancer metastasis. CM is highly malignant and easily metastasizes, thus severely threatening health and vision of most cases. Notably, one of the methylated transferases, METTL3, plays a dominant role in ocular melanoma.^10^ However, the role of METTL14, another important methyltransferase, in CM has not yet been studied. Thus, the expression and function of METTL14 in CM remain unknown, which, given the severity of the disease, warrants further investigation.

In this study, we compared the expression of m6A methyltransferase METTL14 in CM tissues with normal choroidal tissues. The oncogenic role of METTL14 in CM was evaluated both in vivo and in vitro, while the underlying molecular mechanism was clarified by identifying the critical target of METTL14.

## 2 MATERIALS AND METHODS

### 2.1 Specimen preparation and data collection

Tissue samples (both CM and normal choroidal tissues) were collected from 36 volunteer patients admitted to our hospital during October 2016 to December 2021. CM tissues were resected from patients with clinically and pathologically diagnosed CM. In details, inclusion criteria of the patient are shown as below: (1) with high suspicion of choroidal melanoma based on clinical imaging data and clinical ophthalmological examination; (2) absence of other pathologies; (3) receiving enucleation surgery in our hospital, and the patients were diagnosed as choroidal melanoma based on both intraoperative frozen and postoperative pathological examination; (4) intraoperative pathology showed no invasion of the tumours to the optic nerve; (5) postoperative pathological examination also confirmed that there were no tumour cells seen in the optic nerve tissue; (6) the tumours of these patients were all at the intraocular stage. While normal choroidal tissues were excised from patients with mechanical ocular trauma or ocular atrophy. The patient demographics and detailed information are shown in the Table S1 and File S1. All participants provided written informed consent, and ethical approval was issued by the ethics committee of The First Hospital of China Medical University (approval No.: AF‐SOP‐07‐1.1‐01). The samples were divided into two portions, one was soaked in formalin immediately, and the other kept at −80°C.

### 2.2 Western blot (WB) assay

Total protein from tissues or cell lysates was extracted using a buffer containing 1% phenylmethylsulfonyl fluoride (PMSF), and protein concentrations were determined using a bicinchoninic acid (BCA) assay kit (Beyotime Institute of Biotechnology). Protein (40 μg/lane) was denatured and isolated by SDS (10%)‐polyacrylamide (PAL) electrophoresis (140 V, 50 min). The protein was transferred to a polyvinyl formal (PVF) membrane (350 mA, 90 min) blocked with 5% milk (0% fat), which was then sealed in a container at 37 °C for 60 min. The membrane was subsequently incubated with 5% milk (0% fat) containing the following primary antibodies at 4 °C for 12 h: anti‐METTL14 (1:1000, 51104S, Cell Signalling Technology), anti‐runt‐related transcription factor 2 (RUNX2, 1:1000, 12556S, Cell Signalling Technology), anti‐β‐catenin (1:1000, 8480S, Cell Signalling Technology), anti‐phospho‐GSK3β (1:1000, 9322S, Cell Signalling Technology), anti‐GSK3β (1:1000, 9315S, Cell Signalling Technology) and anti‐β‐actin (1:1000, 3700S, Cell Signalling Technology). Membranes were rinsed three times using Tween‐20 buffer for 5 min each and incubated using the corresponding secondary antibodies at 37°C for 1 h. Density was measured using ImageJ software (National Institutes of Health), and protein bands were normalized to β‐actin.

### 2.3 Immunohistochemistry (IHC)

Sections of CM and normal tissue samples were embedded in paraffin, followed by deparaffinization and rehydration. The slides were incubated overnight at 4 °C with the primary antibodies anti‐METTL14 (1:1000, HPA038002, Sigma‐Aldrich) and anti‐RUNX2 (1:1000, MAB2006, RD), followed by addition of secondary antibodies to an avidin‐biotin‐peroxidase complex (e.g. biotinylated anti‐rabbit and anti‐mouse antibodies in goats). The slides were kept at 25°C for 60 min and successively stained with DAB reagent and counterstained with haematoxylin. Images were captured using an inverted microscope (EVOS XL system, AMEX1200; Life Technologies Corp) (magnifications = 200× and 40×).

### 2.4 Cell lines and cell culture

The human CM cell lines OCM1 and MUM‐2B were purchased from the Chinese Academy of Sciences Type Culture Collection Cell Bank. The passage time was ≤180 days with mycoplasma elimination performed by the company. Using six‐well plates, we cultured OCM1 cells in RPMI medium (Hyclone; GE Healthcare) and 10% FBS, and MUM‐2B cells were allowed to proliferate in DMEM medium (Hyclone; GE Healthcare) and 10% FBS. All CM cell lines were maintained at 37°C in 5% CO_2_. At a cell fusion rate of 90%, cells were incubated with trypsin (1 ml) for 5 min and neutralized with growth medium (1 ml). Finally, they were centrifuged and passaged.

### 2.5 siRNA

At a confluence of 60%, the OCM1 and MUM‐2B cells were transfected with METTL14 siRNA (JTS scientific) using Lipofectamine 3000 (Invitrogen). siRNA sequences were as follows (5′–3′): siMETTL14‐1# (sense GGAUGAAGGAGAGACAGAUTT and anti‐sense CCUGGGAAGACUAAGACUUTT), siMETTL14‐2# (sense CAAAGAUGAGCAGAGAGAAAUUGCU and anti‐sense AGCAAUUUCUCUCUGCUCAUCUUUG).

### 2.6 RNA extraction and qRT‐PCR

Extraction of total RNA from OCM1 and MUM‐2B cells was conducted 2 days after siRNA transfection using RNAiso Plus (Takara Biotechnology). cDNA was generated using Prime Script RT Master Mix (Takara Biotechnology), while the SYBR Premix ExTaq™ kit (Takara Biotechnology) was employed for RT‐PCR. A LightCycler 480 II system (Roche) was used for RNA detection with β‐actin as the internal control. The primers for METTL14, RUNX2 and β‐actin were as follows (5′–3′): METTL14 (sense GAACACAGAGCTTAAATCCCCA and anti‐sense TGTCAGCTAAACCTACATCCCTG), RUNX2 (sense GCGCATTCCTCATCCCAGTA and anti‐sense GGCTCAGGTAGGAGGGGTAA), and β‐actin (sense CATGTACGTTGCTATCCAGGC and anti‐sense CTCCTTAATGTCACGCACGAT). The 2^−ΔΔCt^ method was used to determine the relative RNA expression levels.

### 2.7 Transwell assay

Transfected cells (1 × 10^6^) in growth medium (200 μl, containing no serum) were transferred to the upper chamber of a transwell plate, and another 600 μl of the medium was added to the bottom chamber. Cells were incubated at 37°C in 5% CO_2_ for 1 day. Using a swab, we removed residual cells from the upper chamber and fixed the cells by incubation with 4% paraformaldehyde for 600 s. Next, a crystal violet stain was added, and the cells were further incubated for 10 min. Images were collected using an optical microscope and analysed using ImageJ (National Institutes of Health). Additionally, the transwell filter was coated with Matrigel (BD) and dried overnight for invasion assays.

### 2.8 Lentivirus transfection

METTL14 and RUNX2 knockdown and overexpression plasmids were purchased from GeneChem. All the procedures were performed in accordance with manufacturer's instructions. Selection of stable transformants was conducted over two weeks using puromycin (5 μg/ml) and confirmed by WB assay for METTL14 and RUNX2. Cells with confirmed knockdown and overexpression were stored for further tests.

### 2.9 Animal experiments

Female BALB/c nude mice (*n* = 15) aged 4–6 weeks were provided by a commercial contractor and housed in a pathogen‐free environment. In the analysis of METTL14‐mediated metastasis, we injected 150 μl buffer containing 1.0 × 10^5^ MUM‐2B cells transfected with an empty vector (*n* = 5) and shMETTL14 (*n* = 5), respectively, via the lateral tail vein. After 45 days, we excised the lungs to count the metastatic tumours and observed histological sections using haematoxylin and eosin staining. In the analysis of RUNX2‐mediated metastasis, injection of 1.0 × 10^5^ MUM‐2B cells (transfected with empty vector and RUNX2‐knockdown, respectively) in buffer (150 μl) was achieved through the lateral tail vein. Forty‐five days later, lung separation was executed to count the metastatic tumours. All experimental procedures were approved by the ethics committee of The First Hospital of China Medical University (CMU2021090).

### 2.10 Characterization by PET/CT imaging

Tumour‐bearing mice (n = 5 per group), identified using METIS PET, were injected with ~100 μCi [18F] fluoro‐D‐glucose via the tail vein. Anaesthesia was conducted using isoflurane (3% and 2% for induction and maintenance, respectively, in pure O_2_), and mice were placed in a prone position in the centre of the scanner. The obtained PET and CT metadata were reconstructed, and each image was statistically analysed. An organ shape was contoured on each slide, which comprises a part of this organ in the fused PET‐CT image.

### 2.11 The Cancer Genome Atlas (TCGA) data

We conducted a bioinformatic analysis using data from the TCGA database. Survival curves for CM patients with different levels of RUNX2 were selected using a gene expression profiling interactive analysis (GEPIA) (http://gepia.cancer‐pku.cn/) based on a suitable expression threshold.

### 2.12 Methylated RNA immunoprecipitation (MeRIP)

We evaluated the abundance of specific mRNA transcripts in m6A immunoprecipitation and input groups by qPCR using a MeRIP kit (BersinBio). RNA was randomly divided into 100 nucleotide fragments, and immunoprecipitation was performed with anti‐m6A or anti‐rabbit IgG antibodies linked by A/G magnetic beads. A magnetic frame was used to elute the m6A‐precipitated RNA. Enriched RNA was extracted by phenol‐chloroform and ethanol precipitation, and m6A modification towards particular genes was determined by qPCR analysis, using the primer for MeRIP‐qPCR RUNX2‐MeRIP‐3′ UTR: TCCTCTGAAAAGGCAGCAGG; GCATGCCACAGAAGGACTCT. Note that the m6A sites of specific genes were obtained online (http://www.cuilab.cn/sramp).

### 2.13 Plasmid development and dual‐luciferase reporter assay

Cells were seeded into individual wells of a 24‐well plate. Wild‐type/mutated pmiR‐RB‐Report‐RUNX2‐3′‐UTR plasmids (GeneChem) were transfected with METTL14‐OE and vector MUM‐2B cells, respectively. After 48 h, the firefly and Renilla luciferase activities were measured by Dual‐Luciferase Assay kit (Promega). The experimental groups were divided into a RUNX2‐WT + vector, RUNX2‐WT + METTL14‐OE, RUNX2‐Mut + vector and RUNX2‐Mut + METTL14‐OE group.

### 2.14 Statistical analysis

All tests were executed in triplicates, and all data were described as mean ± standard deviation (SD). Software GraphPad Prism of version 8.0 was employed for statistical analysis. Inter‐group differences were assessed using a Student's *t*‐test. The differences in expression levels between paired samples were assessed using a Wilcoxon signed‐rank test. Survival rates were estimated using the Kaplan–Meier log‐rank method.

## 3 RESULTS

### 3.1 METTL14 is overexpressed in CM tissues

**FIGURE 1:**
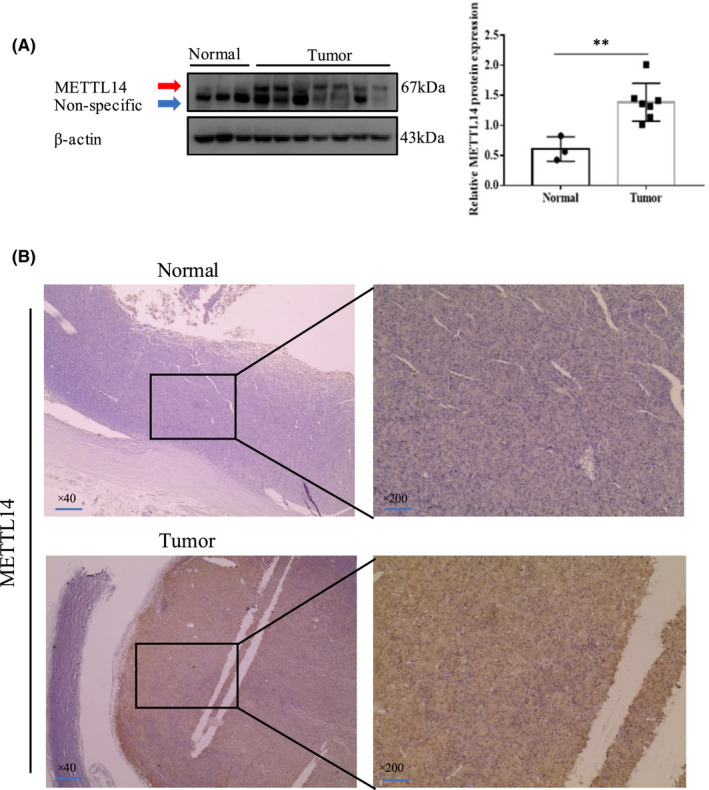
METTL14 overexpression in choroidal melanoma CM tissues. (A) Protein expression of METTL14 in CM and normal choroidal tissues. (B) Immunohistochemistry (IHC) staining of METTL14 in CM and normal choroidal tissues.

The results of WB assay revealed that the protein of METTL14 was drastically upregulated in CM tissues than that in normal choroidal tissues (Figure 1A). Expressions of METTL14 in CM and normal choroidal tissues were detected by IHC assays. According to Figure 1B, the METTL14 expression in the CM tissues was drastically upregulated in comparison with that in the normal choroidal tissues. Overall, METTL14 played an important role in CM progression.

### 3.2 METTL14 promotes migration and invasion of CM cells both in vivo and in vitro

**FIGURE 2:**
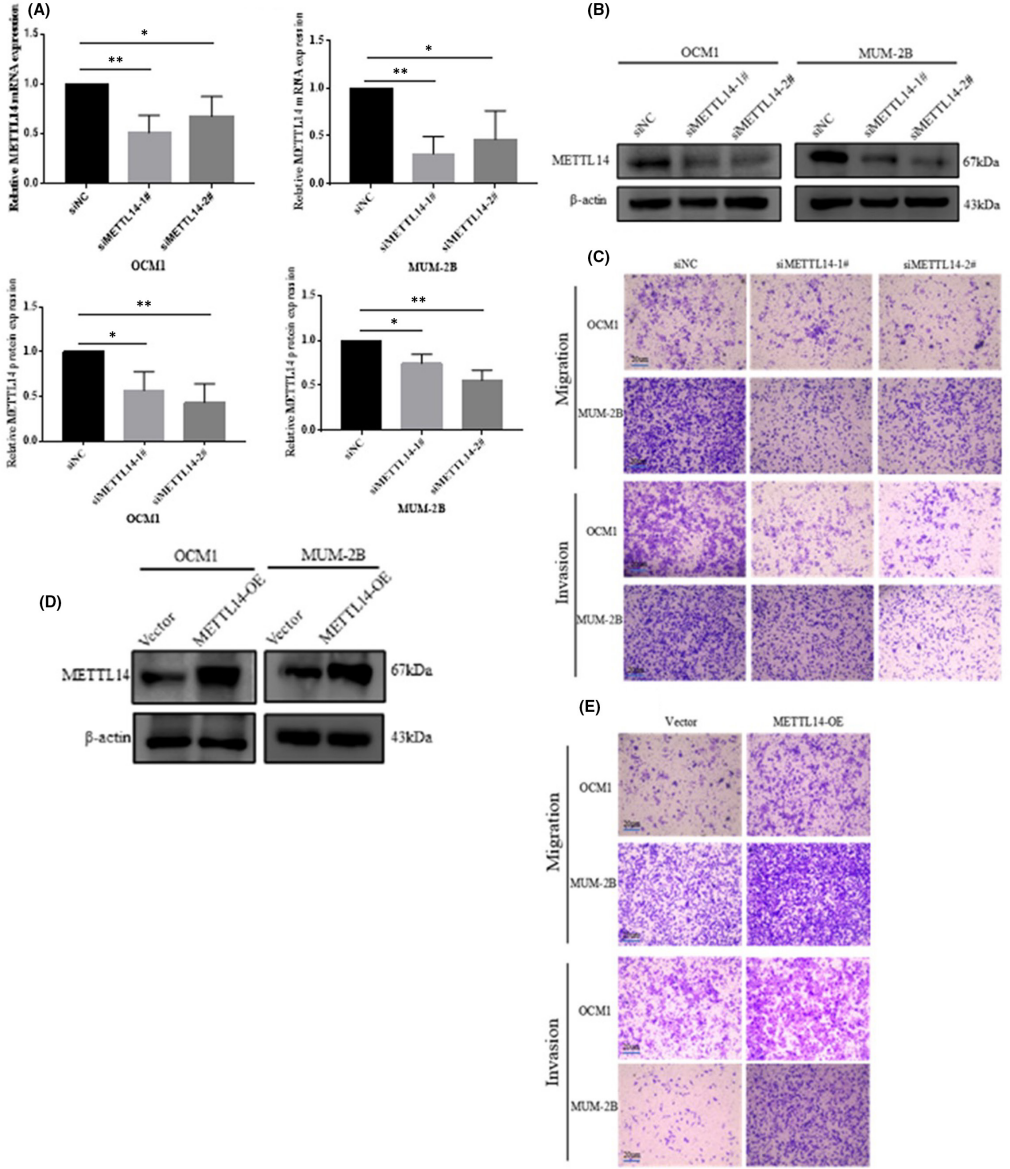
METTL14 promotes migration and invasion of CM cells in vitro. (A) Expression efficiency of METTL14 after transfection with siNC or siMETTL14 in OCM1 and MUM‐2B cells. (B) Western blot (WB) analysis was performed to measure METTL14 protein levels in OCM1 and MUM‐2B cells transfected with siMETTL14/siNC. (C) Transwell assays were performed to determine the effects of METTL14 on migration and invasive capability in OCM1 and MUM‐2B cells. Magnification, 100×. Data are presented as mean ± SD of three independent replicates. Student's *t*‐test was used to analyse inter‐group differences. (D) WB analysis was performed to measure METTL14 protein levels in OCM1 and MUM‐2B cells transfected with empty vectors and METTL14 overexpression lentivirus. (E) Transwell assays were performed to determine the effects of METTL14 on migration and invasive capability in OCM1 and MUM‐2B cells.

**FIGURE 3:**
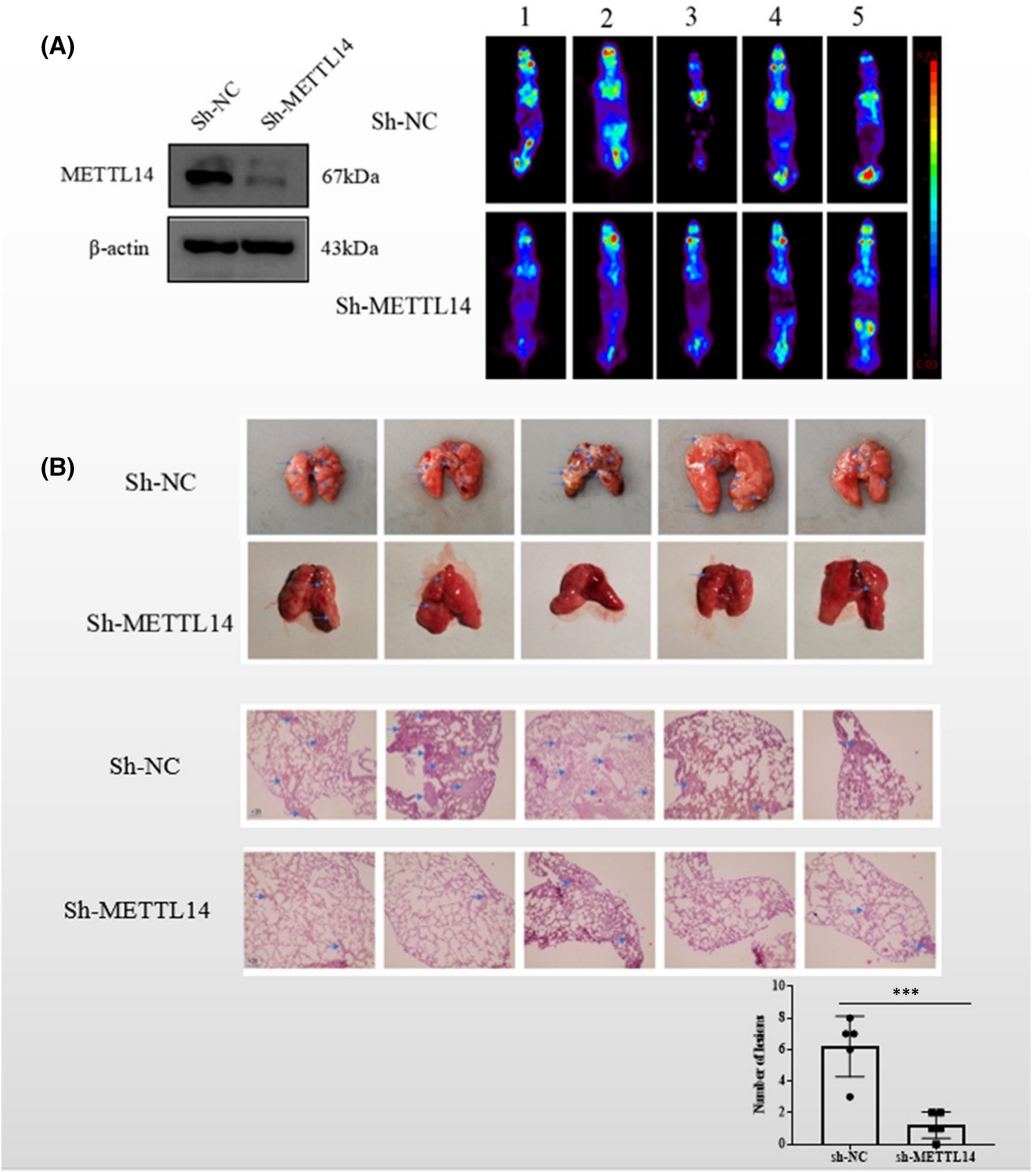
METTL14 promotes migration and invasion of CM cells in vivo. (A) Whole‐body coronal PET images of mice with tumours were obtained 45 days after the tail vein injection of CM cells. WB analysis verified the successful construction of knockdown stabilized MUM‐2B cells. (B) WT and METTL14 knockdown MUM‐2B stable cells were injected via the tail vein, respectively. Haematoxylin and eosin (H&E) staining results and metastatic lung tumours are shown, and the lung tumour counts are shown in the bar graph. The blue arrows indicate the sites of metastatic tumour.

To assess the role of METTL14 in CM, METTL14 was knocked down in human CM cell lines; qRT‐PCR revealed that METTL14 mRNA was downregulated as a result of transfection with si‐METTL14‐1 and si‐METTL14‐2 (Figure 2A). Additionally, WB analysis revealed that METTL14 protein levels were downregulated as a result of transfection (Figure 2B). In the transwell assays, METTL14 depletion markedly reduced the migratory and invasive abilities of OCM1 and MUM‐2B (Figure 2C). The WB analysis demonstrated that METTL14 protein levels were upregulated as a result of transfection with overexpressed METTL14 lentivirus (Figure 2D). Consistently, with the overexpression of METTL14, migration and invasion of CM cells increased significantly (Figure 2E). Thus, METTL14 enhanced the migration and invasion of CM cells in vitro. To clarify the biological functions of METTL14 in vivo, the empty vector and METTL14‐knockdown MUM‐2B cells were injected for 45 days, followed by lung colonization. It was demonstrated the number of metastatic tumours in the lungs in the METTL14‐knockdown group showed decreased tendency, suggesting that METTL14 can enhance the metastasis of CM cells both in vitro and in vivo (Figure 3A,B).

### 3.3 METTL14 activates the Wnt/β‐catenin signalling pathway (Wβ‐CSP) in CM cells

**FIGURE 4:**
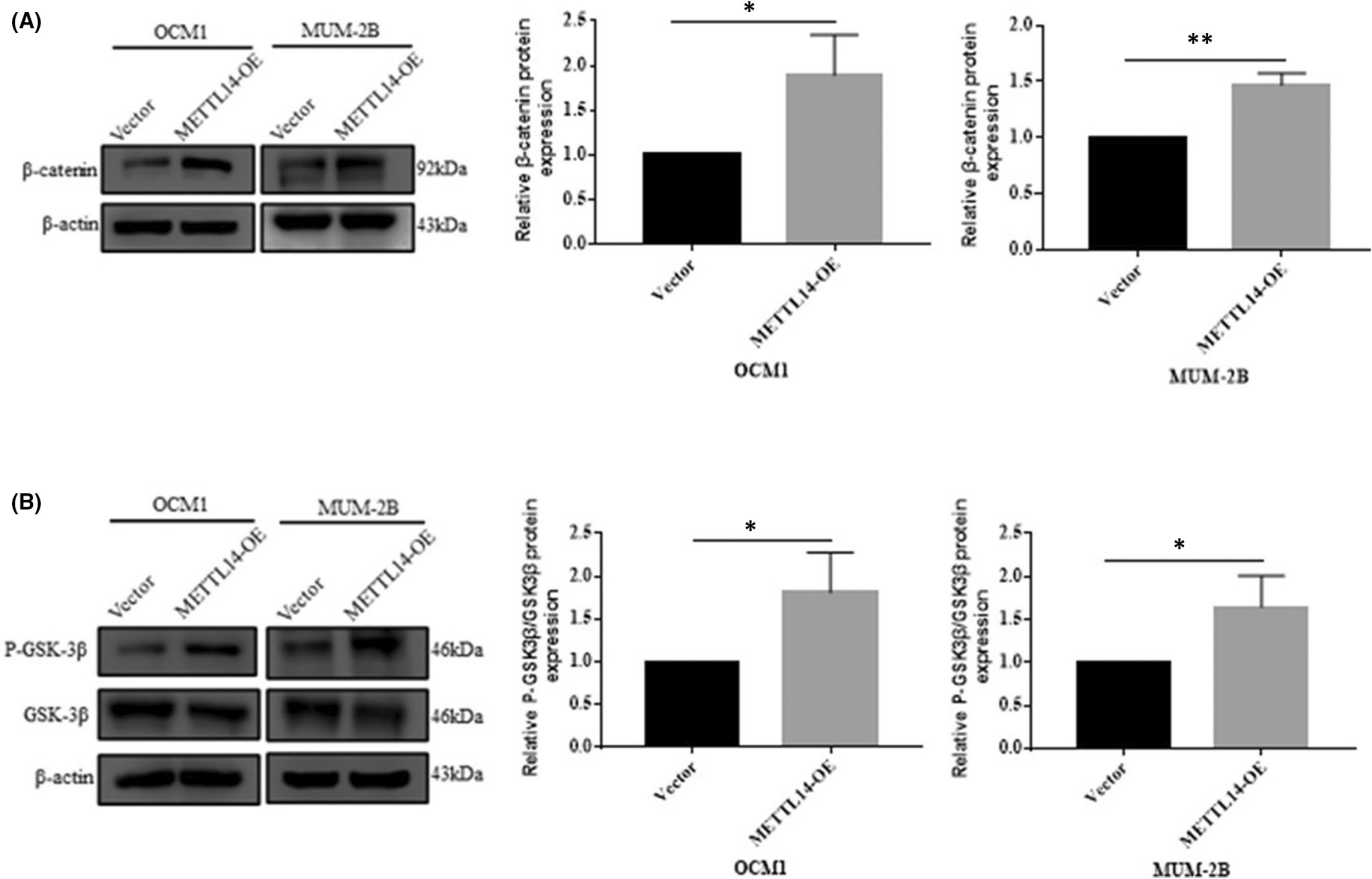
METTL14 activates the Wnt/β‐catenin signalling pathway (Wβ‐CSP) in CM cells. (A) The β‐catenin expression of the Wnt signalling pathway was examined by WB analysis in METTL14 stably‐transfected OCM1 and MUM‐2B cells. (B) The expression of critical members of the Wnt signalling pathway was validated by WB in METTL14 stably‐transfected OCM1 and MUM‐2B cells.

The mechanism of METTL14 in cell migration and invasion was further explored. The Wβ‐CSP regulates cell migration, invasion and metastasis in various tumours, including CM.^11^, ^12^, ^13^ Following METTL14 upregulation, β‐catenin protein levels were upregulated (Figure 4A). P‐GSK3β protein levels were upregulated without significantly affecting total GSK3β protein expression, which confirms activation of the Wβ‐CSP (Figure 4B).

### 3.4 RUNX2 is an essential METTL14 target gene in CM

**FIGURE 5:**
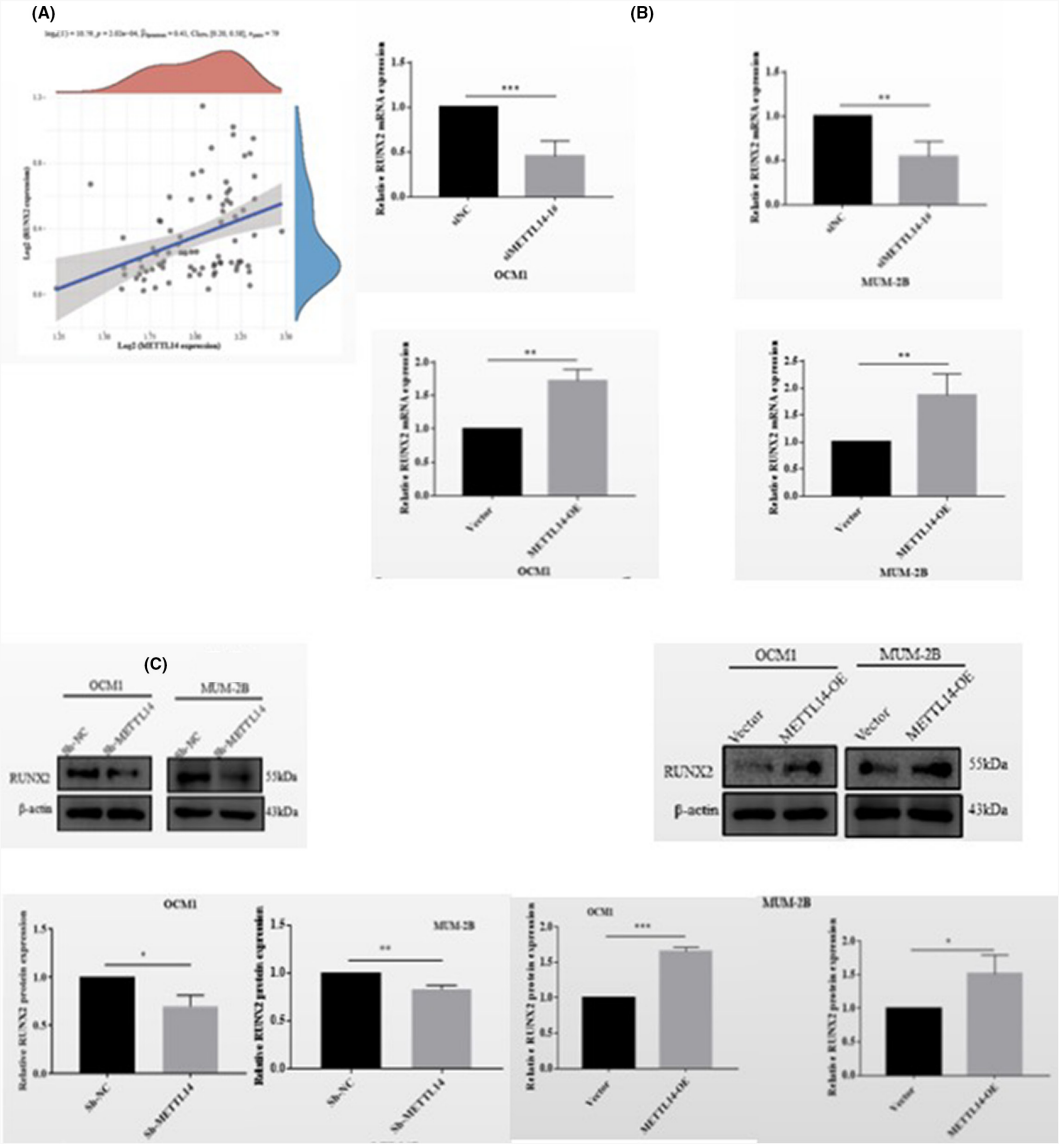
RUNX2 is the key target of METTL14 in CM. (A) Correlation of METTL14 and RUNX2 expression in the TCGA database. (B) qRT‐PCR analysis of RUNX2 mRNA after METTL14 inhibition or overexpression. (C) WB results indicate that protein expression of RUNX2 is significantly downregulated or upregulated after METTL14 knockdown or overexpression, respectively.

**FIGURE 6:**
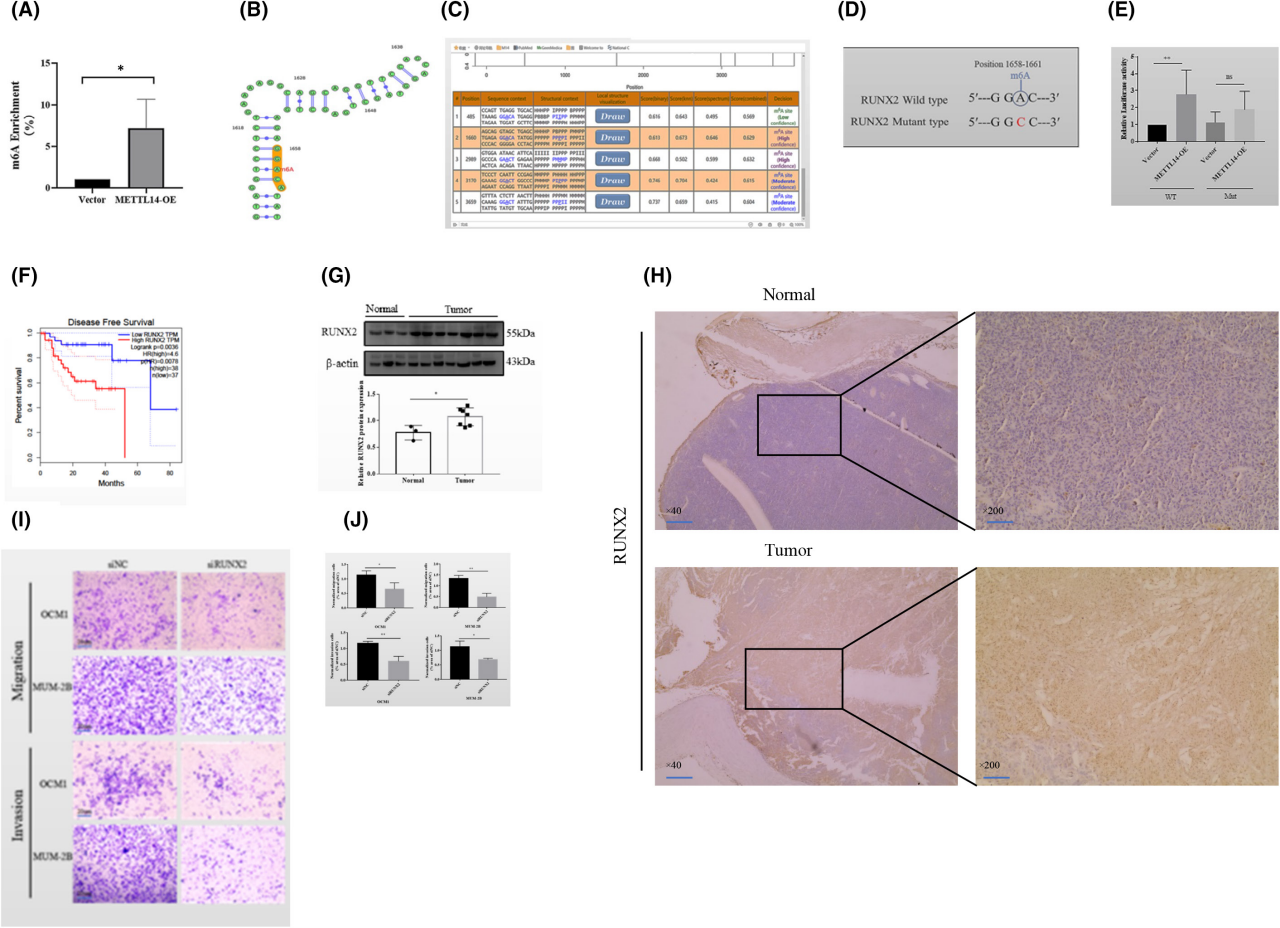
RUNX2 mRNA can be methylated by METTL14 and it promotes migration and invasion of CM cells in vitro. (A–E) RUNX2 mRNA can be methylated by METTL14. A, MeRIP‐qPCR analysis was used to determine m6A modification enrichment in RUNX2 mRNA after overexpressing METTL14 in MUM‐2B cells. (B) C, RUNX2 methylation site. (D) Mutations at the RUNX2 methylation site. (E) Luciferase activities in MUM‐2B cells transfected with RUNX2‐WT or RUNX2‐Mut + vector or METTL14. (F–J) RUNX2 promotes migration and invasion of CM cells in vitro. (F) Kaplan–Meier survival analysis of CM tumour samples suggest that high RUNX2 expression levels are related to reduced overall survival (OS). (G) Protein expression of RUNX2 in CM and normal choroidal tissues. (H) IHC staining of RUNX2 in CM and normal choroidal tissues. (I, J) Transwell assays were used to determine the effects of RUNX2 on migration and invasion capability in OCM1 and MUM‐2B cells.

The expression of transcription factor RUNX2 is upregulated in melanoma cells^14^ and mediates cell migration and invasion in many cancers, including prostate, colorectal and gastric cancer.^15^, ^16^, ^17^ However, the expression of RUNX2 has not been described for CM and RUNX2 potentially has m6A sites.^18^ In the TCGA database, METTL14 expression was positively correlated with RUNX2 expression (Figure 5A), suggesting a potential positive regulatory mechanism. RUNX2 mRNA and protein were down‐ and upregulated upon depletion and overexpression of METTL14, respectively (Figure 5B,C). Moreover, MeRIP‐qPCR confirmed that METTL14 overexpression enhanced m6A enrichment of RUNX2 mRNA (Figure 6A). According to data from m6A databases, SRAMP was located at the base of the 3′‐UTR; 1660A is the m6A motif; and GGAC in the base sequence was mutated to GGCC. A schematic diagram showing the methylation site on RUNX2 and mutation at this site is presented in Figure 6B–D. The RUNX2 3′‐UTR‐reporter luciferase assay revealed that overexpression of METTL14 enhanced the luciferase activity of constructs in the wild‐type RUNX2 3′‐UTR, but not in the mutated RUNX2 3′‐UTR sequence (Figure 6E). It is also demonstrated that RUNX2 mRNA can be methylated by METTL14.

### 3.5 RUNX2 affects migration and invasion of CM cells in vitro and knockdown of RUNX2 inhibits lung metastasis in nude mice models

**FIGURE 7:**
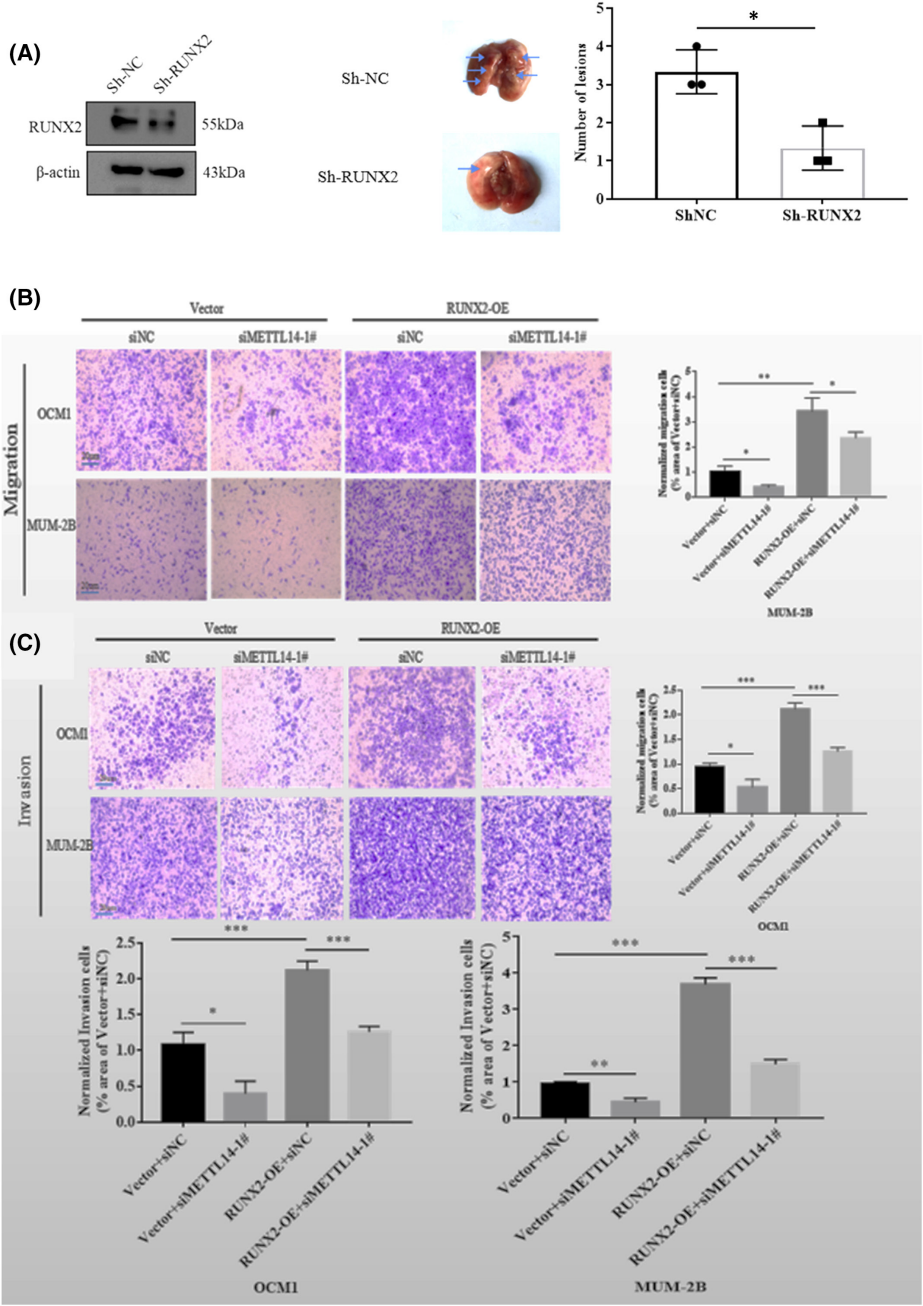
Role of METTL14 and RUNX2 in metastasis. (A) Knockdown of RUNX2 inhibits lung metastasis in nude mice models. WB verified the successful construction of knockdown stabilized MUM‐2B cells. WT and RUNX2 knockdown MUM‐2B stable cells were injected via the tail vein. Representative images of metastatic lung tumours. (B, C) Transwell assay demonstrates the effects of METTL14 and RUNX2 on the migration and invasion capability of CM cells. Overexpression of RUNX2 partially reversed the reduction in cell migration and invasion caused by decreased METTL14 expression. All data are presented as mean ± SD from three independent replicates. Student's *t*‐test was used to assess inter‐group differences.

Low overall survival was associated with high levels of RUNX2 (Figure 6F). The protein expression of RUNX2 in CM tissues was upregulated compared to normal choroidal tissues (Figure 6G). According to the IHC assay, RUNX2 expression in CM tissues was upregulated compared to normal choroidal tissues (Figure 6H). The transwell assay revealed that the depletion of RUNX2 reduced invasion and migration of OCM1 and MUM‐2B (Figure 6I,J). To clarify the biological function of RUNX2 in vivo, empty vector and RUNX2‐knockdown MUM‐2B cells were injected in BALB/c mice. After 45 days, the lungs in the RUNX2‐knockdown group had a lower number of metastatic tumours compared to the empty vector group (Figure 7A). The results confirm the oncogenic role of RUNX2 in CM.

### 3.6 RUNX2 overexpression partially restores invasion and migration of cells induced by decreased METTL14

We performed transwell assays to evaluate the effects of RUNX2 overexpression in cell invasion and migration ability under reduced levels of METTL14. RUNX2 overexpression partially restored the migration and invasion abilities of CM cells (Figure 7B,C), suggesting that RUNX2 is a dominant effector by which METTL14 promotes migration and invasion.

## 4 DISCUSSION

CM is a complex disease with high rates of metastasis, and no effective therapies have been described to date.^10^ m6A is known to affect pathological processes, as well as physiological functions, particularly during tumorigenesis,^19^, ^20^ with m6A dysregulation observed in multiple cancers.^6^, ^21^, ^22^ It has been demonstrated that abnormal epigenetic regulation of gene function via m6A modification plays a crucial role in human tumorigenesis and cancer progression.^6^, ^9^, ^23^ METTL14 is involved in the dynamic and reversible process of m6A modification.^24^ METTL14 regulates the initiation and progression of multiple cancers by acting as both oncogene and tumour suppressor gene; however, METTL14 can act as a promoter in some types of cancer. For instance, METTL14 upregulation promotes PERP mRNA N6‐adenosine methylation, facilitating metastasis and growth in pancreatic cancer.^25^ METTL14 has a positive effect on migration and invasion of breast malignancy cells by regulating the expression of m6A and hsa‐miR‐146a‐5p.^26^ Thus in breast malignancies, the upregulation of m6A in peripheral blood could serve as a novel biomarker, while METTL14 upregulation has an improved diagnostic role in peripheral blood screening.^27^ As a cancer suppressor, METTL14 inhibited the progression of colorectal malignancies by modulating the processing of m6A‐dependent primary miR‐375^28^; m6A modification of circORC5 mediated by METTL14 suppressed the progression of gastric malignancies by regulating the miR‐30c‐2‐3p/AKT1S1 axis^29^; METTL14 can suppress proliferation and metastasis of colorectal cancer by downregulating oncogenic long non‐coding RNA XIST^30^; and the metastatic potential of hepatocellular carcinoma was suppressed by METTL14 by modulating the processing of primary microRNA, which is dependent on m6A.^31^

The function of some m6A components has been reported in CM. For instance, METTL3, an m6A ‘writer’, was downregulated in ocular melanoma tissues,^10^ and m6A demethylation of FOXM1 mRNA mediated by ALKBH5 was associated with uveal melanoma (UM) progression.^32^ This study provides evidence that METTL14 is overexpressed in CM tissues, promotes migration and invasion of CM cells in vitro and enhances tumour metastasis in vivo. Nevertheless, the role of METTL14 in other CM phenotypes (e.g. proliferation and drug resistance) requires further investigation. The Wβ‐CSP regulates cell migration, invasion and tumour metastasis in various tumours, including CM.^11^, ^12^, ^13^ For example, overexpression of microRNA‐130a suppressed the migration and invasion of UM cells by downregulating USP6 to inactivate the Wβ‐CSP.^11^ It blocks the pathway and inhibits growth, migration and invasion of cancer cells.^33^ METTL14 expression is positively correlated with m6A modification.^7^ It is reported in the literature that enhanced m6A modification in different cancer types activates the Wnt/β‐catenin signalling pathway.^34^ In addition, inhibiting METTL14 by adenosylhomocysteine (SAH) could reduce the intracellular kinase activity of GSK3β.^35^ In consistence with these observations, our results showed that enhanced METTL14 in cancer cell lines indeed activated Wnt/β‐catenin signalling pathway and elevated phosphorylated GSK3β. Overall, our results suggest that METTL14 activates the Wβ‐CSP in CM cells. A previous study reported that the expression of METTL3 was comparatively reduced in ocular melanoma tissues.^10^ Our study demonstrated that METTL14 favours migration and invasion of CM cells, which is not contradictory to the activity of METTL3; for example, METTL3 and METTL14 can play opposing roles in the regulation of hepatocellular carcinoma.^36^ Ours is the first study to demonstrate that METTL14 has an oncogenic role and favours migration and invasion as a tumour promoter in CM, and thus could serve as a novel biomarker.

RUNX2 is a key regulator of osteogenesis and essential for normal skeletal and bone development.^37^ RUNX2 expression is tightly regulated in normal osteoblastic cells.^38^, ^39^, ^40^ In details, expression of RUNX2 protein is dramatically high in G0 stage, low at G1/S transition, low during G2 and M phases, and high in early G1 stage.^39^, ^40^ RUNX2 favours a quiescent state and inhibits cell proliferation. Abnormal overexpression of RUNX2 might promote its role in oncogenesis. RUNX2 is highly expressed throughout the cell cycle in osteosarcoma cells. Overexpression of RUNX2 hinders cell cycle entry in S phase and inhibits cell proliferation in pre‐osteoblasts, leading to abnormal osteogenic differentiation, which eventually progress to osteosarcoma.^41^

METTL14 overexpression promoted m6A modifications at RUNX2 3′‐UTR, facilitating the migration and invasion of CM cells. This reveals a novel aspect of epigenetic alterations associated with CM and provides promising targets for novel interventional therapies. RUNX2 upregulation favours the migration of T‐ALL cells and progression in leukaemia,^42^ while it regulates progression in renal cell carcinoma.^43^ In addition, FTO regulates RUNX2 mRNA expression via its demethylation of m6A.^18^ In the present study, RUNX2 served as an essential target gene for METTL14 in CM and was dependent on m6A. The role of RUNX2 in tumour metastasis, development and progression has been widely reported. Since the first report describing the role of RUNX2 in the differentiation and migration of osteoblasts into chondrocytes and its engagement in the proinvasive/promigratory behaviour of various tumour cells, especially in melanoma cells, RUNX2 has been established as a key factor in metastasis.^44^ In our study, overexpression of RUNX2 partially restored cell invasion ability under reduced levels of METTL14 expression, suggesting that RUNX2 is a dominant effector by which METTL14 enhances invasion of CM cells. Here, we report the first evidence of RUNX2 as an oncogene in CM and propose its use as a novel biomarker. However, further investigation is required to determine whether other downstream factors of METTL14 are involved in CM.

In conclusion, METTL14 promotes migration and invasion of CM cells by targeting RUNX2 mRNA. Our findings elucidate the molecular pathogenic mechanisms of CM and provide a basis for developing novel therapeutic strategies for CM by targeting m6A regulators.

## AUTHOR CONTRIBUTIONS

**Xi Zhang:** Conceptualization (lead); data curation (lead); formal analysis (lead); investigation (lead); methodology (lead); visualization (equal); writing—original draft (lead). **Xiaonan Zhang:** Investigation (equal); methodology (equal). **Tengyue Liu:** Investigation (equal); methodology (equal). **Zhe Zhang:** Data curation (equal); formal analysis (equal). **Chiyuan Piao:** Data curation (equal); formal analysis (equal). **Hong Ning:** Funding acquisition (lead); project administration (lead); resources (lead); supervision (lead); writing—review and editing (supporting).

## FUNDING INFORMATION

This study was funded by the Natural Science Foundation of Liaoning Province (grant No. 20180551171). Major Social Development Project of Provincial Science and Technology Agency, Grant/Award Number: 2020JH1/10300002

## CONFLICT OF INTEREST

The authors confirm that there are no conflicts of interest.

## CONSENT FOR PUBLICATION

Written informed consent for publication was obtained from all participants.

## Supporting information


File S1

Table S1

## Data Availability

The datasets generated/analysed in the present study are available upon reasonable request from the corresponding author.
