# Supplementary material for: METTL14 promotes migration and invasion of choroidal melanoma by targeting RUNX2 mRNA via m6A modification
Source: J Cell Mol Med. 2022 Oct 20;26(22):5602–13. doi: 10.1111/jcmm.17577 (PMC9667526; doi:10.1111/jcmm.17577)
Supplement: Supplementary file 2 — Table S1 [file JCMM-26-5602-s001.docx]

Supplementary Table 1. Patient demographics

|  | **Normal tissue (n=28)** | **Tumor tissue (n=36)** |
| --- | --- | --- |
| Gender | Male 10; Female 18 | Male 17; Female 19 |
| Age (y) | 40 ±20 | 64 ± 9 |
